# Identification of G protein-coupled receptors in *Schistosoma haematobium* and *S. mansoni* by comparative genomics

**DOI:** 10.1186/1756-3305-7-242

**Published:** 2014-05-27

**Authors:** Tulio D L Campos, Neil D Young, Pasi K Korhonen, Ross S Hall, Stefano Mangiola, Andrew Lonie, Robin B Gasser

**Affiliations:** 1 Faculty of Veterinary Science, The University of Melbourne, Parkville, Victoria, Australia; 2 FIOCRUZ, Centro de Pesquisas Aggeu Magalhães, Recife, Pernambuco, Brazil; 3 Victorian Life Sciences Computation Initiative (VLSCI), The University of Melbourne, Parkville, Victoria, Australia; 4 Institute of Parasitology and Tropical Veterinary Medicine, Berlin, Germany

## Abstract

**Background:**

Schistosomiasis is a parasitic disease affecting ~200 million people worldwide. *Schistosoma haematobium* and *S. mansoni* are two relatively closely related schistosomes (blood flukes), and the causative agents of urogenital and hepatointestinal schistosomiasis, respectively. The availability of genomic, transcriptomic and proteomic data sets for these two schistosomes now provides unprecedented opportunities to explore their biology, host interactions and schistosomiasis at the molecular level. A particularly important group of molecules involved in a range of biological and developmental processes in schistosomes and other parasites are the G protein-coupled receptors (GPCRs). Although GPCRs have been studied in schistosomes, there has been no detailed comparison of these receptors between closely related species. Here, using a genomic-bioinformatic approach, we identified and characterised key GPCRs in *S. haematobium* and *S. mansoni* (two closely related species of schistosome).

**Methods:**

Using a Hidden Markov Model (HMM) and Support Vector Machine (SVM)-based pipeline, we classified and sub-classified GPCRs of *S. haematobium* and *S. mansoni*, combined with phylogenetic and transcription analyses.

**Results:**

We identified and classified classes A, B, C and F as well as an unclassified group of GPCRs encoded in the genomes of *S. haematobium* and *S. mansoni*. In addition, we characterised ligand-specific subclasses (i.e. amine, peptide, opsin and orphan) within class A (rhodopsin-like).

**Conclusions:**

Most GPCRs shared a high degree of similarity and conservation, except for members of a particular clade (designated SmGPR), which appear to have diverged between *S. haematobium* and *S. mansoni* and might explain, to some extent, some of the underlying biological differences between these two schistosomes. The present set of annotated GPCRs provides a basis for future functional genomic studies of cellular GPCR-mediated signal transduction and a resource for future drug discovery efforts in schistosomes.

## Background

Diseases caused by parasites inflict major socio-economic impact worldwide, particularly in developing countries. For instance, schistosomiasis affects more than 200 million people, and 600 million are at risk of contracting disease in endemic areas [1-3]. Schistosomiasis is caused by blood flukes (schistosomes; Phylum Platyhelminthes; class Trematoda). *Schistosoma haematobium* and *S. mansoni* are two main causative agents of this disease in humans, predominantly in Africa [4]. As there is no vaccine against schistosomiasis, treatment relies almost exclusively on the use of one drug, praziquantel [5]. With concerns regarding the emergence of praziquantel resistance in schistosomes, there is a need to search for alternative, effective compounds [6,7].

Schistosomes, including *S. haematobium* and *S. mansoni*, have complex, aquatic life cycles, involving snails as intermediate hosts [4]. After leaving snails in water, larvae (cercariae) infect humans by penetrating skin. The ensuing schistosomules migrate via the bloodstream to the lung and then toward the liver, where they develop to adults and mate. Subsequently, adult couples migrate to their final destination to reproduce. *S. mansoni* migrates to the mesenteric venules and the portal system, eggs pass into the liver or through the intestinal wall and are then excreted in the faeces. Conversely, *S. haematobium* migrates to the vessels of the urinary bladder, where females produce eggs that pass through the bladder wall and are released in urine. Eggs of both schistosome species hatch in freshwater and infect an intermediate, snail host; *S. haematobium* prefers snails of the genus *Bulinus*[8], whereas *S. mansoni* prefers *Biomphalaria*[9]. Pathological changes in the human host arise when eggs become entrapped in tissues, causing granulomata and subsequent fibrosis [10]. Symptoms of chronic schistosomiasis include fatigue, malnutrition, diarrhoea, anaemia and/or severe abdominal pain [11]. Chronic *S. haematobium* infection is known to be associated with bladder cancer [12,13] and can predispose to HIV/AIDS [14]. Although biological differences exist between these closely related species, some morphological and life history strategies should be relatively conserved, including processes involved in receiving endogenous and exogenous molecular signals.

Schistosomes rely on conserved signal transduction pathways for a broad range of cellular processes, such as mating, reproduction, nutrient recognition and uptake as well as host responses [15-17]. Current evidence indicates that environmental signals are transduced from the external surface of the tegument [18]. The tegument of trematodes is rich in excretory/secretory (ES) inclusions, bounded externally by a plasma membrane bearing a dense glycocalyx, and is composed of conserved proteins, suggesting similarities in the structure and function of the surface layer [19]. Proteomic and functional expression analyses [20-23] have identified various salient, molecular components of the tegumental matrix, including G protein-coupled receptors (GPCRs).

GPCRs are the largest transmembrane (TM) protein superfamily of eukaroytes, and are responsible for detecting many extracellular signals and transducing them to the heterotrimeric G proteins, which then communicate with various downstream effectors, including key molecules involved in developmental and/or neuromuscular functions [24]. A salient, usually conserved feature of GPCRs is their seven inter-membrane, anti-clockwise alpha helices, each containing 25 to 35 amino acid residues. GPCRs have been explored as drug targets, because of their diversity and essential biological roles, and it is estimated that 30-40% of the current pharmaceuticals available today target these receptors [25]. Well-characterized ligands that bind to GPCRs include neurotransmitters, odorants, pheromones and hormones. This interaction produces signals that are transduced into the cell, activating, via G-proteins, specific intracellular events. Based on their functional similarity or homology, the GPCR superfamily is usually divided into six main classes: A (rhodopsin-like), B (secretin receptor family), C (metabotropic glutamate/pheromone), D (fungal mating pheromone receptors), E (cyclic AMP receptors) and F (frizzled/smoothened) [26], although other classification systems, such as GRAFS, have also been proposed [27]. Among the known classes of GPCRs, the large group of class A (rhodopsin-like) receptors, particularly the amine subclass, are recognised as targets for the development of novel drugs [28,29].

High throughput genomic sequencing, increased computing power and better bioinformatic tools have enhanced the *in silico* characterization and annotation of GPCRs of metazoan organisms [30-41]. Extensive diversity within the GPCR family poses a challenge for the identification and classification of receptors from divergent species [42]. To overcome this challenge, pipelines have been proposed or established for GPCR identification and classification from inferred proteomes using machine learning techniques, such as Hidden Markov Models (HMMs) [43] and Support Vector Machines (SVMs) [44]. Using this approach, platyhelminth GPCRs have been identified and characterized for *S. mansoni* and the free-living planarian *Schmidtea mediterranea*[45]*.* However, in the latter study, the lack of genetic similarity between these two species and the fragmented nature of the *S. mediterranea* genome limited the characterisation of GPCRs in each species. To address this, herein, we undertook a comprehensive study of GPCRs in two closely related parasitic trematodes, employing well-assembled draft genomes. Since GPCR families are diverse both functionally and structurally, there is a need to identify and classify receptors from flatworms with confidence, particularly if the goal is to search for new drug targets. Logically extending a previous investigation [45], we (i) employed an improved bioinformatic approach for the identification and classification of GPCRs in *S. haematobium* and *S. mansoni*, two closely related species of schistosome [46], (ii) undertook a detailed exploration of members of class A (rhodopsin-like), and (iii) discussed the findings in the context of functional genomics and drug discovery.

## Methods

### Inferred protein sequences and GPCR data sets for training

Amino acid sequences were conceptually translated from genes of *S. haematobium*[47] and *S. mansoni*[48,49]. With the exception of class F, training and reference sequences encoding GPCRs were obtained from a public GPCR database (GPCRDB) [50], including 35829 class A (rhodopsin-like), 1969 class B (secretin-like), 1701 class C (metabotropic glutamate/pheromone), 337 vomeronasal receptors (V1R and V3R), 8 class E (cAMP) and 721 taste receptors (T2R); 588 class F (frizzled) sequences were obtained from the Pfam database [51]. Sequences in GPCRDB are classified using the International Union of Pharmacology (IUPHAR) system [52]. Sequences with discrepancies in description or family classification in GPCRDB and without experimental support of functionality were removed. Experimentally validated GPCRs of *S. mansoni* and molluscs, including *Aplysia californica*, *Lymnaea stagnalis* and *Spisula solidissima*[22,23,53-59], were added to the data set.

### Prediction of TM domains and construction of Hidden Markov Models (HMMs) and Support Vector Machine (SVM) protein classifiers

TM domains were predicted for each protein sequence in the GPCR training sets and from those inferred for *S. haematobium* and *S. mansoni* using TOPCONS-single [60]. Custom Python scripts were written to parse results generated by TOPCONS-single, and also to extract and concatenate transmembrane (cTM) domain sequences.

For validation purposes, human sequences were removed from the GPCRDB-derived data set used for subsequent HMM training. The cTM domain sequences of each GPCR training set were aligned using the program MAFFT [61], converted to the Stockholm format and an HMM was built for each GPCR class using hmmbuild [62]. The quality of the cTMD alignments and HMM models was assessed by determining whether the GPCR training sequences from each class were accurately identified by their respective HMM using hmmsearch [62]. In addition, GPCRs predicted from the human proteome were compared with those from the ENSEMBL database [63] to assess HMMs. The sensitivity and specificity of GPCR prediction were assessed by conducting an area-under-the-curve (AUC) analysis based on expected and observed predictions, also considering the proportions of false-positive and false-negative results [64].

Sub-classification of the class A GPCRs was performed using a “one-against-one” approach [65]: one for the 19 class A subclasses (SVM1), and another for the 7 class A-amine-subclasses (SVM2). Each SVM was generated using the program LIBSVM [66]. For SVM1, TM domains were extracted and concatenated for each subclass within class A. Fixed-length, dipeptide frequency vectors were calculated for each cTM domain using an available Perl script [45]. For the purpose of training SVM1, GPCRs classified as class A were divided into training (20%) and test (80%) subsets using the subset.py script in LIBSVM [66], ensuring that each subset included an even proportion of each GPCR subclass. For SVM2, dipeptide frequency vectors were calculated from full-length amino acid sequences, and 5-fold cross-validation was applied. The script easy.py in LIBSVM was used for the optimum selection of the kernel parameters, employing a grid space and applying data-scaling as well as 5-fold cross-validation. The most accurate parameters from the cross-validation steps were used for SVM training.

### Identification, classification and sub-classification of schistosome GPCRs

The cTM domains extracted from the inferred proteomes of *S. haematobium* and *S. mansoni* were used to classify or subclassify GPCRs. Classification was inferred using hmmsearch (E-value cut off: < 0.01) to identify the most homologous GPCR HMM model for each cTM. Dipeptide composition vectors were then created for individual class A GPCRs, which were then classified based on their predicted ligand specificity using the SVM1 model. Rhodopsin-like (class A) GPCRs predicted to bind an amine ligand were classified further using a second SVM model (SVM2). Prior to SVM classification, each GPCR data set was first scaled using LIBSVM [66], with the parameters defined by each SVM training optimization process, inferred using the program Python v.2.6 and an available Python script (scale.py) [66]. Putative GPCRs were then examined for the presence of non-GPCR protein sequences based on amino acid sequence homology (BLASTp, E-value cut-off: < 0.00001) to proteins in InterProScan (including Pfam), ChEMBL, GPCR SARfari, KEGG, Pfam, SwissProt and TrEMBL databases [51,67-70]. Sequences with significant homology to non-GPCR proteins of other organisms were removed.

### Phylogenetic analysis

Trees were constructed for each class A subclass and also for classes B and F. Putative GPCRs containing 5–8 TM domains and identified by HMM models in both *S. haematobium* and *S. mansoni* were grouped according to their corresponding GPCR classes/subclasses, and then aligned using the program PRALINE [71]. The program PRALINE [71] was used to progressively align amino acid sequences using PSI-BLAST (3 iterations; E-value cut-off: < 0.01; Protein Data Bank, PDB), integrating secondary structural information predicted using the program PSIPRED [72] as well as TM information employing the program PHOBIUS [73] and the BLOSUM62 amino acid scoring matrix, with fixed gap opening (12) and extension (1) penalties. The final alignment was improved using the program MUSCLE employing the *-refine* option [74].

The final, predicted GPCR data sets were each subjected to phylogenetic analysis by Bayesian inference (BI) in MrBayes v.3.2 [75], employing the Whelan and Goldman model [76] and using the final 75% of 100000 iterations to construct a 50% majority rule tree, with the nodal support for each clade expressed as a posterior probability value (pp). The BI analysis was run until the potential scale reduction factor (PSRF) was approximately 1. Phylogenetic trees were drawn using the program FigTree v.1.4 (http://tree.bio.ed.ac.uk/software/figtree/).

If no orthologous sequence was initially detected in the heterologous species of schistosome, genomic, transcriptomic and proteomic datasets were scrutinized, employing the programs BLAT [77], tBLASTn [78] and BLASTp [78], respectively, until an ortholog was found. In the absence of a complete match using available protein sequence data sets, the sequence was inferred based on the conceptual reverse translation of the protein to nucleotide sequence and alignment to a genomic scaffold (including 10000 bp up- and down-stream) using BLAT [77]. This genomic region was then exhaustively searched for a predicted coding domain matching the missing protein using the program Exonerate [79], employing the multi-pass suboptimal alignment algorithm and the protein2genome:bestfit model.

### Analysis of transcription

Following the selection of GCPRs, levels of transcription were explored in the adult stages of *S. haematobium* and *S. mansoni* using available RNA-seq data [47,49]. These data were filtered for quality (PHRED score of >30) using Trimmomatic [80] and aligned to the final nucleotide domains encoding the GPCR orthologs identified here using the Burrows-Wheeler Alignment (BWA) tool [81]. For each RNA-seq library, reads that mapped to individual coding domains were counted using SAMtools [82]. For each data set, levels of transcription were normalised and expressed as reads per kilobase per million mapped reads (RPKM) [83]. For each GPCR, a relative measure of transcription in the adult stage was inferred by ranking individual genes from *S. haematobium* and *S. mansoni* by their RPKM values (highest to lowest). The top 25% of genes were defined as having very high transcription (*S. haematobium* RPKM range: 62 to 18765; *S. mansoni*: 68 to 16368), and 26-50% as high (*S. haematobium*: 18 to 62; *S. mansoni*: 20 to 68), 55-75% as medium (*S. haematobium*: 3 to 18; *S. mansoni*: 4 to 20), and 75-100% as low (*S. haematobium*: 0.02 to 3; *S. mansoni*: 0.03 to 4).

## Results

### Improvements and validation of HMMs for GPCR classification

A consensus approach was used to identify TM domains in proteins submitted to GPCRDB. These domains were extracted and concatenated for each sequence. For every GPCR class, TM domain sequences (with the exclusion of those predicted from human proteins) were then aligned. Due to substantial sequence variation among representatives of class A in GPCRDB, cTMs of each of the 19 subclasses of class A were aligned separately. HMMs constructed for each set of aligned cTMs were reliable, with >95% (E-value cut-off: <0.01) of GPCRDB-classified proteins being correctly assigned to their original category using the trained HMMs.

As GPCRs of humans are best characterized structurally, functionally and as drug targets [25,84,85], we used these GPCRs to validate our HMM approach. Using our HMMs to interrogate the human proteome, there was no evidence of false-positive results. The calculated AUC value of >99% demonstrated a high specificity and sensitivity of prediction. We were also able to predict all human GPCR sequences that had been removed from the training set. Of the predicted human GPCRs with at least one GPCR Pfam domain (1701), ~90% (1523) were predicted to possess between 5 and 8 TM domains; thus, we defined this range as a “gold standard” filter for predicting membrane-spanning proteins in the schistosomes studied.

### GPCRs encoded in the *S. haematobium* and *S. mansoni* genomes belong to classes A, B, C and F

**Figure 1:**
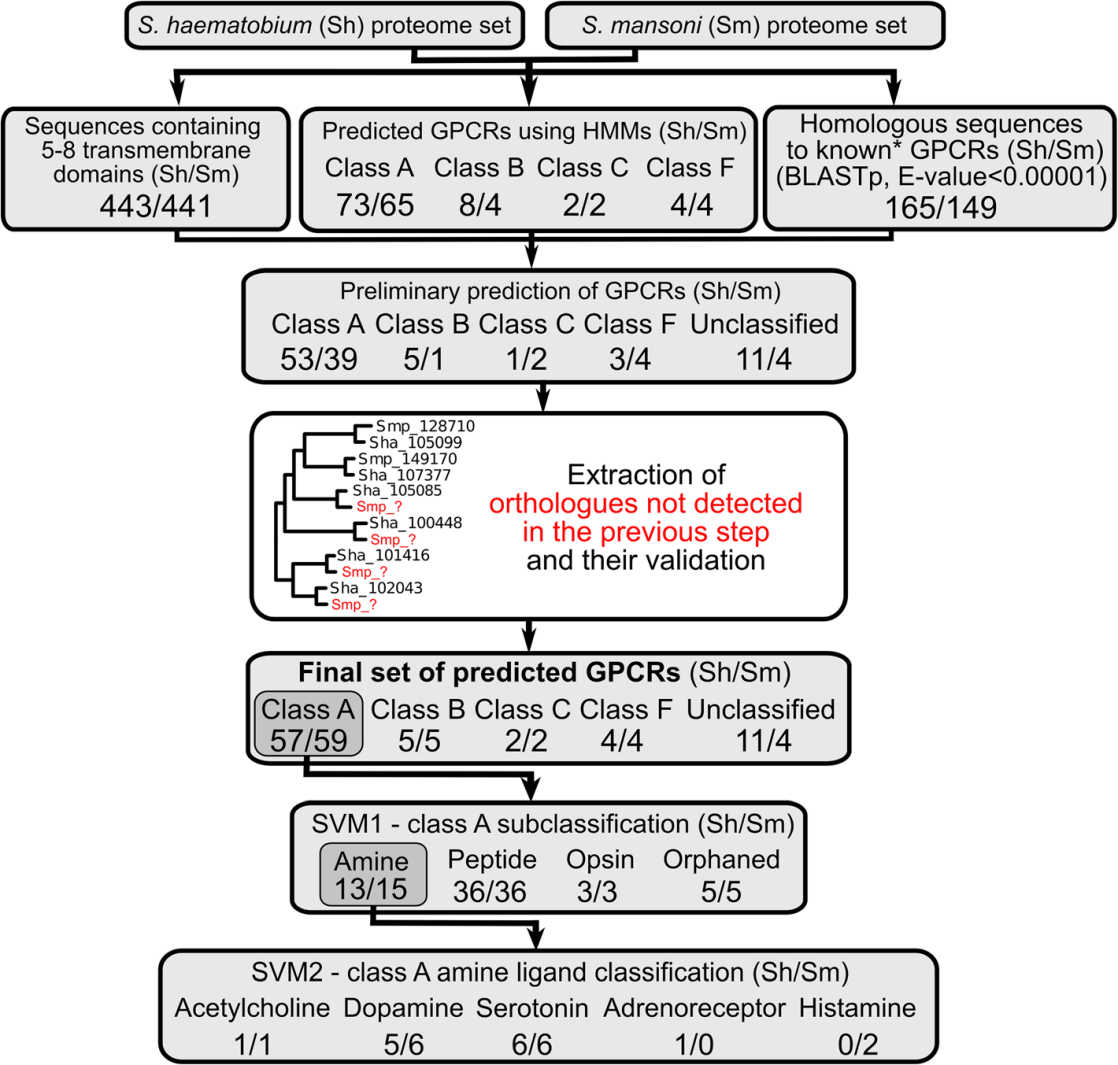
**Summary of results for the identification and classification of GPCRs in *Schistosoma haematobium *and *S. mansoni.*** Top to bottom: First, from the inferred proteomes [47,49], the numbers of sequences with transmembrane (TM) domains, the numbers of GPCRs of each class predicted by Hidden Markov Models (HMMs) and the numbers of significant matches to known* GPCRs (from databases such as SwissProt, TrEMBL and KEGG) are presented. Second, the preliminary sets of GPCRs categorised to the class level are shown, after filtering sequences that did not contain 5–8 transmembrane domains. Third, orthologs not detected in published gene sets were identified in the phylogenetic trees generated (paired one-to-one orthologs were expected for the two closely related schistosome species). Fourth, the final sets of GPCRs for each class, including the numbers of sequences found by homology but not predicted by HMMs, are shown. Finally, the numbers following sub-classification by SVM1 (class A subclass) and SVM2 (class A amine sub-classification – ligand affinity) are given.

From the inferred proteomes of *S. haematobium/S. mansoni*, 443/441 sequences were predicted to contain TM domains (Figure 1). Based on amino acid sequence homology (E-value cut-off: <0.00001), 165/149 *S. haematobium/S. mansoni* proteins shared significant homology to annotated GPCRs in public databases. In total, only 31 GPCRs from *S. haematobium* and 27 from *S. mansoni* did not share significant amino acid sequence homology to manually-curated proteins in the SwissProt database, indicating a sequence divergence from organisms other than trematodes.

Using our HMMs, GPCRs including 73/65 class A, 8/4 class B, 2/2 class C, and 4/4 class F (Figure 1) were identified in *S. haematobium/S. mansoni*. Additionally, we found 11/4 sequences with significant homology (E-value cut-off: <0.00001) to GPCRs that had not been predicted by our HMMs. These latter sequences might not have been identified due to significant divergence from the HMMs. Classification and further sub-classification of schistosome GPCRs was performed only on proteins detected by HMMs and predicted to encode 5 to 8 TM domains. Using these stringent criteria, 53/39 class A, 5/1 class B, 1/2 class C and 3/4 class F GPCRs were identified in *S. haematobium/S. mansoni*.

**Figure 2:**
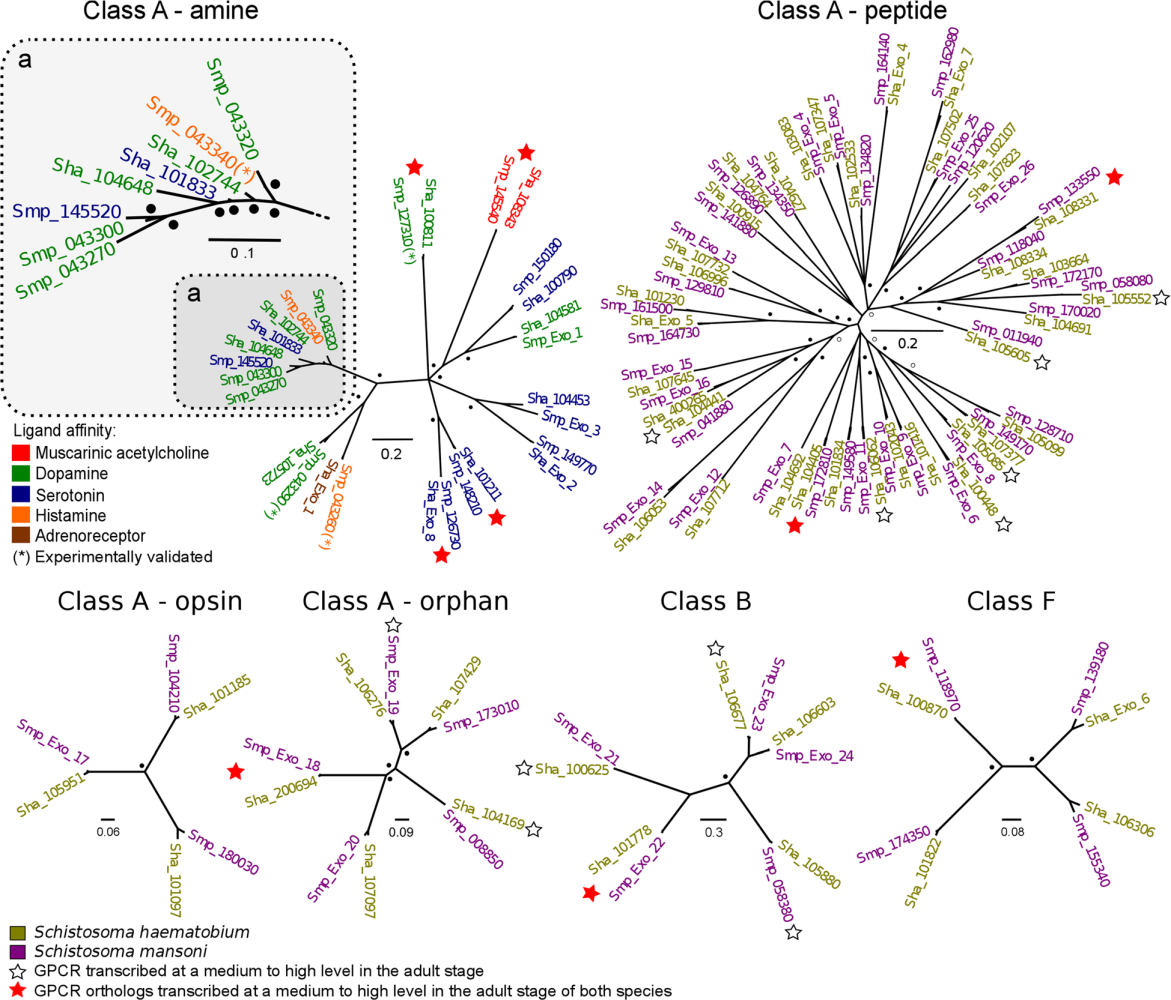
**Phylogenetic trees displaying the relationships of GPCRs representing subclasses of class A, and also classes B and F identified in *Schistosoma haematobium *and *S. mansoni.*** In each tree, the amino acid changes per site are shown. The posterior probability (pp) of each node is indicated by small circles (pp = 0.7-0.8) or dots (pp > 0.8) at nodes. Medium to high transcription (cf. Methods section) in the adult stages of the schistosomes is identified by asterisks. The class A amine tree shows the SVM sub-classification, coloured according to ligand affinity. Asterisks indicate experimentally validated GPCRs. Relationships of the sequences representing the SmGPR clade [23] (inset a shows an enlargement). Shae and Smp are codes for sequences from *S. haematobium* and *S. mansoni*, respectively.

Exhaustive searches were conducted in heterologous schistosome genomes to identify any one-to-one GPCR orthologs that were absent from published gene sets (Figure 1). Matching genomic regions and coding domains were identified, and protein sequences conceptually translated. By comparing corresponding orthologs, we also detected three incorrect intron-exon boundaries (within genes represented by codes Smp_160020, Sha_107760 and Sha_100228) that needed correcting. In total, 26 GPCR sequences were retrieved using this approach; these sequences were then annotated and added to the data set (see Additional file 1: Table S1). Phylogenetic trees were constructed using this final set of predicted GPCRs (Figures 1 and 2). In the predicted proteomes of *S. haematobium*/*S. mansoni*, 53/59 class A (rhodopsin-like), 5/5 class B (secretin-like), 2/2 class C (metabotropic glutamate/pheromone) and 4/4 class F (frizzled) GPCRs were identified. These numbers correspond to ~ 5% of the total number of sequences of the inferred proteomes. GPCRs representing other classes were not detected. The final GPCR-coding domains, their classification and homology search results were compiled (see Additional file 1: Table S1).

In most cases, predicted GPCRs were identified in both *S. haematobium* and *S. mansoni*, with the exception of proteins classified within the amine subclass of class A GPCRs containing a previously characterized *S. mansoni*-specific (SmGPR) clade [23] (Figure 2). For this group, we identified paralogous sequences (Sha_101833 and Sha_104648; Smp_043270, Smp_145520, Smp_043300 and Smp_043320), possibly resulting from gene duplication (Figure 2). Four of the *S. haematobium*-specific sequences grouped with SmGPRs (Figure 2). However, unlike the other classified GPCRs, these molecules did not group in a pairwise, orthologous manner. The class A amine tree displaying the SmGPR sequences is consistent with a previously published dendogram [23], suggesting that SmGPRs share a common basal group, and have diverged in these two schistosome species, with paralogs Smp_043260, Sha_Exo1, Smp_043290 and Sha_105723 diverging first.

### SVM-based sub-classification and analysis of class A GPCRs reveal distinct differences between *S. haematobium* and *S. mansoni* in the amine subclass

We applied the strategy of dipeptide composition frequencies for the GPCR sub-classification employing SVMs using an established method [44]. To enhance the specificity of our SVMs, potentially misclassified sequences in GPCRDB were removed and experimentally validated GPCRs from molluscs added. GPCRs of molluscs were included, as these invertebrates are taxonomically related to schistosomes and belong to the Super-phylum Lophotrochozoa [86].

Class A GPCR sequences identified by HMMs were sub-classified by SVM1. After parameter training and optimization, both SVMs were able to classify >95% of the validated sets correctly. SVM1 sub-classification identified 13/15 amine, 36/36 peptide, 3/3 (rhod)opsin and 5/5 orphans among the class A GPCR sequences (Figure 1). A phylogenetic tree was constructed for each subclass of class A, and also for classes B and F (Figure 2); a tree was not constructed for class C due to the small number of sequences identified (Additional file 1: Table S1).

Of all subclasses within class A, the peptide ligand subclass is the most abundant for schistosomes. This subclass mainly represents proteins involved in neuropeptidergic signalling, which is essential for parasite development and survival [87]. Based on annotation (see Additional file 1: Table S1), peptide ligand GPCRs showed homology to known neuropeptides, such as neuropeptide F (NPFs) and neuropeptide Ys (NPY) characterized in other organisms, whereas some of them appear to be schistosome-specific [88]. The opsin ligand subclass included GPCRs that were inferred to be involved in photoreception, converting photons of light into electrochemical signals [89] (see Additional file 1: Table S1). Finally, an orphan group of proteins was identified; these proteins had significant sequence homology to class A rhodopsin-like receptors but not to other known subclasses. The orphan GPCRs identified are likely to be flatworm- or schistosome-specific.

**Figure 3:**
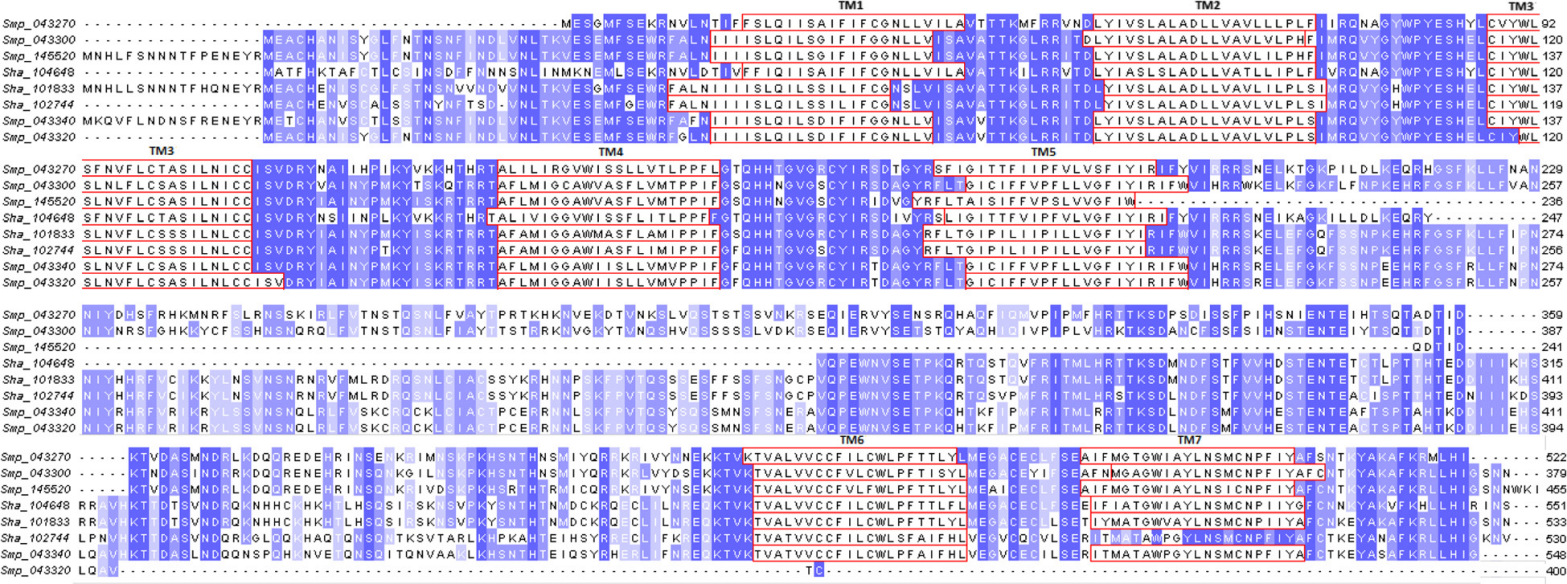
**Alignment of sequences representing the SmGPR clade (cf. Figure**2**).** Conserved, transmembrane (TM) domains are outlined in red; the most conserved amino acid residues in other sequence regions are in blue, with the least conserved residues in palest. Most sequences within this clade were predicted, using a Support Vector Machine (SVM), to bind to dopamine, with the exception of those with extended N-termini.

For the amine subclass, the SVM2 model was used to infer ligand affinity for 5/6 dopamine, 6/6 serotonin and 1/1 acetylcholine GPCRs in *S. haematobium/S. mansoni* (Figures 1 and 2; Additional file 1: Table S1). In addition, two *S. mansoni* GPCRs (encoded by genes Smp_043340 Smp_043260) were classified as histamine receptors (Additional file 1: Table S1). Interestingly, based on SVM2 classification, one *S. haematobium* protein (encoded by Sha_Exo_1) was classified as an adreno-receptor but was orthologous to the Smp_043260 histamine receptor (Figure 2), which suggests orthologs may bind different ligands. This was also observed for sequences within the SmGPR clade (Figure 2), which showed variation in ligand specificity, despite their high amino acid sequence similarity (37-86%; mean: 62%) upon pairwise comparison.An alignment was made of sequences representing the SmGPR clade, and the TM domains were identified (Figure 3). In total, five sequences within the clade were predicted as being dopamine-responsive. In addition, two receptors were predicted to bind serotonin and one to histamine; these protein sequences had extended N-termini, which was a remarkable difference compared with those inferred to bind dopamine. Although conservation was observed within the TM domains among all sequences represented in the SmGPR clade, there was considerable sequence variation (mean of 50%) in the intracellular loop between TM domains 5 and 6 (see Figure 3).

### Transcription analysis of GPCRs indicated active orthologs in the adult stage

Of the 70 GPCRs identified in *S. haematobium*, five, 14 and 46 were transcribed at high, medium and low levels, respectively; five did not have RNA-seq support (see Additional file 1: Table S1). Of the 68 GPCRs identified in *S. mansoni*, two, 10 and 44 were transcribed at high, medium and low levels, respectively; 12 did not have RNA-seq support (see Additional file 1: Table S1). One-to-one orthologs with high or medium levels of transcription are indicated in the phylogenetic trees (Figure 2). In the amine, peptide and orphan subclasses (class A) and the class B tree, four, two, respectively one and two orthologs of *S. haematobium* and *S. mansoni* exhibited medium to high levels of transcription. In addition, 10 GPCRs from *S. haematobium,* and two from *S. mansoni* displayed medium to high transcription levels, in contrast to their corresponding orthologs (Figure 2). Sequences in the SmGPR clade did not represent genes with high transcription.

## Discussion

The availability of genomic and transcriptomic data sets for schistosomes [48,49,90] provides unprecedented opportunities to explore GPCRs that are essential for parasitic flatworm life and survival. Here, subsets of GPCRs encoded in the genomes of *S. haematobium* and *S. mansoni* were predicted and classified with a high degree of confidence. Although the method established here is similar to a previous approach [45], it applies stricter criteria to remove false-positive results, incomplete sequences and includes transcriptional evidence of gene prediction, thus increasing specificity. Our approach improved the classification of class A receptors by creating HMMs for each subclass within this class, instead of relaxing the level of significance of the HMMs. A “gold standard” was also applied to filter sequences according to the number of TM domains. Comparing the two closely related species, *S. haematobium* and *S. mansoni*, at the genomic level improved GPCR annotation by identifying orthologs missing from published gene sets and enhanced structural prediction of genes.

The GPCR repertoires of *S. haematobium* and *S. mansoni* are conserved, except for members of the SmGPR clade, which appear to have diverged in these species. The paralogs in the SmGPR clade might result from gene duplication events or mutations that alter ligand affinity. These differences might explain, to some extent, some of the underlying biological differences between *S. haematobium* and *S. mansoni*. Interestingly, the experimentally validated receptors SmGPR-1 and SmGPR-2, which are responsive to histamine, have been reported as expressed in the peripheral nervous system (PNS) and the suckers of adult *S. mansoni*[58]. In addition, SmGPR-3, being responsive to dopamine, is highly expressed in the central nervous system (CNS) of this schistosome in both larval and adult stages, and has also been detected in PNS and suckers of adult worms [23]. Given the divergence in amino acid sequence and sub-classifications of GPCRs within each *S. haematobium* and *S. mansoni*, further study of their functional differences between these species is warranted. Importantly, the bioinformatic pipeline used here was able to correctly classify all experimentally validated GPCRs of *S. mansoni* studied to date [22,23,58] and might thus be applied to GPCRs of other metazoan parasites.

In the present study, 13 and 15 proteins responsible for biogenic amine signalling were predicted for *S. haematobium* and *S. mansoni*, respectively. Receptors of the biogenic amine subclass (class A) are of significant biological interest, because they are known to be responsible for several modulations in neuromuscular function, including metabolic activity, movement and muscle contraction [91-93]. The activity of these receptors is highly likely to be essential for parasite survival inside the host. This subclass includes small molecules, such as acetylcholine, serotonin (5-hydroxytryptamine: 5HT), histamine, catecholamines (adrenaline, noradrenaline and dopamine) and also invertebrate-specific ligands, such as octopamine and tyramine. Depending on the neurotransmitter, they can either stimulate or inhibit neuromuscular or metabolic activity [91]. Serotonin is known to stimulate muscular activity, whereas dopamine causes muscular relaxation in schistosomes [56,94-96]. For these reasons, biogenic amines are well recognised as anthelmintic drug targets [97], and could be the focus of future studies.

As adult schistosomes establish within the vasculature system of the human host, they are the ideal developmental stage to target for treatment [98]. In this study, a number of GPCRs were identified as being transcribed in the adult stages of both *S. haematobium* and *S. mansoni*. Despite the importance of SmGPRs as potential drug targets [58], based on RNA-seq data, none of the SmGPR gene homologs were amongst the GPCR-encoding genes most abundantly transcribed in the adults of the two schistosomes studied. Although transcription has been investigated only in the adult stage to date, SmGPR members might also have key roles in other developmental stages. High coverage RNA-seq [99] should be used to explore the transcription of GPCRs in all developmental stages of these schistosomes; this information might be used to prioritise GPCR drug target candidates.

As praziquantel is the sole drug widely used in millions of people against schistosomiasis, efforts are required to develop new anti-schistosomal drugs, because of concerns of anti-praziquantel resistance in schistosomes. GPCRs have been shown to be valuable drug target candidates in some organisms, but key functional mechanisms of members of this complex superfamily still require detailed investigations [84]. Because membrane proteins are unstable [100], tertiary structures of only a small number of GPCRs have been solved to date using X-ray crystallography; clearly, such structures underpin drug discovery. This is why advanced *in silico* approaches are needed to predict, comprehensively characterize and classify GPCRs based on genomic, transcriptomic and proteomic data. Other bioinformatic tools might be applied to predict receptor structures and drug screening [101]. Despite the challenges of identifying and classifying GPCRs, repertoires have been defined for various organisms, including members of the Platyhelminthes [45]. However, functional validation is still required to support most predictions. Although evidence indicates that the number of predicted GPCRs in GPCRDB is inflated, the addition of experimentally-validated sequences from different groups of organisms will likely contribute to enhancing the prediction of machine learning models that rely heavily on GPCRDB to build sets for training, validation and testing.

## Conclusions

In conclusion, the present study provides a useful resource for the selection of high-priority candidates for functional genomic and biological studies as well as drug target discovery in schistosomes. Methods, such as RNA interference (RNAi) [102,103], can now be used for the functional validation of GPCR-encoding genes in *S. haematobium* and *S. mansoni*. In addition, immuno-molecular methods are already in use for the identification of GPCR ligands and their localization in flatworms [22,23,58]. Using these tools, future insights into the roles of GPCRs in signal transduction, development, reproduction and nutrient uptake in these schistosomes could provide a path to understanding the molecular biology of these worms and parasite-host interactions, and might underpin the design of new interventions. Clearly, this study provides GPCR data for schistosomes that will assist future investigations on both fundamental and applied levels. Improved annotation of GPCRs from other schistosomes might also foster broader comparative investigations.

## Abbreviations

GPCR: G protein-coupled receptors; GPCRDB: GPCR database; HMM: Hidden Markov Model; IUPHAR: International Union of Pharmacology; RPKM: Reads per kilobase per million mapped reads; SVM: Support Vector Machines; TM: Transmembrane.

## Competing interests

The authors declare that they have no competing interests.

## Authors’ contributions

Conceived and designed the study and supervised the project: NDY and RBG. Undertook the study and data analysis: TDLC, NDY and RBG. Contributed to analysis using various tools: PKK, RH, SM and AL. Wrote the paper: TDLC, NDY and RBG. All authors read and approved the final version of the manuscript.

## Authors’ information

Tulio D. L. Campos and Neil D. Young are joint first authors.

## Supplementary Material

Additional file 1: Table S1 Salient characteristics of the GPCRs predicted for *Schistosoma haematobium* and *S. mansoni*, including their classification and protein homology to annotated proteins or conserved domains listed in current, public databases. Click here for file
